# Supplementary material for: Is minor surgery safe during the COVID-19 pandemic? A multi-disciplinary study
Source: PLoS One. 2021 May 11;16(5):e0251122. doi: 10.1371/journal.pone.0251122 (PMC8112651; doi:10.1371/journal.pone.0251122)
Supplement: Attachment — Submitted filename: Response to reviewers.docx [file pone.0251122.s002.docx]

*Dear Robert Jeenchen Chen,*

*We are truly honored of the interest that the Editorial team of PLOS One has paid to our work.*

*We would like to thank the editor their thoughtful comments and constructive suggestions, which helped us to improve the quality of this report. Please find enclosed our revised manuscript and the rebuttal letter that specifically addresses every point they raised.*

*Sincerely yours,*

*Dr Michael Baboudjian*

**COMMENTS FOR THE AUTHOR**

**Editor:**

The scope of "surgery" is ill-defined. Did it include open heart surgery, brain surgery, and some other major surgery? Or just urology surgery? Please specify upfront in the Title.

We thank the editor for this justified comment. This part has been completed in the Methods Section as follow:

“The patients were operated on in one of the hospital's seven surgical departments: urology, digestive, plastic, gynecological, otolaryngology, gynecology or maxillofacial surgery. »

Thus, cardiovascular, thoracic, orthopedic and brain surgeries were not included in this study.

In the Methods, the IRB is only approved in urology, not other surgical specialty. Was it legal to include non-urology surgery cases?

The institutional review board approval from our hospital (Ap-Hm: Assistance Publique des Hôpitaux de Marseille) was provided for all surgical departments, not only for urology. In addition to the IRB, we have requested additional approval from the ethics committee of the French urology association. This is an ethics committee like any other, which can give its approval for a study that does not include only urology patients.

Please specify the scope of "surgery" in the Methods. Open heart surgery? TAVR? TEVAR? EVAR? Brain surgery, Whipple surgery, Bentall surgery, type-A aortic dissection, lung cancer surgery, eye surgery, dental surgery,...? "Surgery" needs to be defined and specified in this manuscript!

As previously described, this part has been completed in the Methods Section as follow:

“The patients were operated on in one of the hospital's seven surgical departments: urology, digestive, plastic, gynecological, otolaryngology, gynecology or maxillofacial surgery. »
