# Supplementary material for: Is minor surgery safe during the COVID-19 pandemic? A multi-disciplinary study
Source: PLoS One. 2021 May 11;16(5):e0251122. doi: 10.1371/journal.pone.0251122 (PMC8112651; doi:10.1371/journal.pone.0251122)
Supplement: Attachment — Submitted filename: Response to reviewers.docx [file pone.0251122.s003.docx]

*Dear Robert Jeenchen Chen,*

*We are truly honored of the interest that the Editorial team of PLOS One has paid to our work.*

*Please find enclosed our revised manuscript and the rebuttal letter that specifically addresses every point they raised.*

*Sincerely yours,*

*Dr Michael Baboudjian*

**COMMENTS FOR THE AUTHOR**

1. Not only in "reply", please also define and specify the specialties/scopes of "surgery" in "Title", "Abstract", and "Methods" in the main text. The revision fails to show it. For example, please add "minor elective surgery" or modify otherwise because it does not include cardiac, neuro-, emergent, or other major surgical cases.

This comment is relevant. In fact, in our hospital there is no cardiac, vascular or neurological surgery. However, some of the patients included underwent major surgery such as cancer surgery (eg laryngectomy, cystectomy, gastrectomy) or kidney transplantation (n=15). In addition, as shown in Table 1, 28 included patients were acute surgical emergencies. Thus, all types of surgery were included: minor and major, elective and urgent surgery.

However, due to the lack of available resuscitation bed during the pandemic period, all surgeries were scheduled to not use postoperative resuscitation care and therefore, were mainly minor surgeries. As suggested by the editor, some changes have been added in the Title, the Abstract and The Manuscript:

Title: Is Minor Surgery Safe During the COVID-19 Pandemic? A Multi-Disciplinary Study

Abstract: Our study included all adult patients who underwent minor surgery in one of the seven surgical departments of our hospital: urology, digestive, plastic, gynecological, otolaryngology, gynecology or maxillofacial surgery.

Methods: The patients were operated on in one of the hospital's seven surgical departments: urology, digestive, plastic, gynecological, otolaryngology (two departments), gynecology or maxillofacial surgery. In our hospital, there was no cardiac, vascular, neurological or orthopedic surgery.

Discussion (Limitations): Finally, the present study reports on a very heterogeneous surgical population and covers a wide range of planned elective surgeries and a variety of emergency surgical presentations. However, our study population consisted mainly of patients who underwent minor elective surgery. This observation should be related to a significant decrease in acute surgical emergencies as observed in previous studies found in the literature [26]. In addition, major surgeries such as cardiac, vascular or neurological surgeries requiring postoperative resuscitation care were not included in our series.

2. In the revised Methods, you specified "all seven surgical departments...." What are these seven surgical departments are unknown to readers.

This comment is justified. As suggested by the reviewer, this data has been added in the Methods section:

“All of the seven surgical departments in our hospital were involved in our study: urology, digestive, plastic, otolaryngology, gynecology or maxillofacial surgery.”

3. Your results and conclusion clearly do not apply to cardiac, vascular, orthopedic, or other major surgical cases (I myself is a cardiac surgeon so I care if your research applies to my specialty or not.). Please specify minor surgery upfront in Title and Abstract.

This is a justified comment. As suggested, we specified in the title and the abstract that the surgeries were mainly minor.

4. Did your cases include emergent or trauma cases?

Yes, some cases were acute surgical emergencies (n=28, 5.1% of all cases, Table 1). These patients therefore represent a very small part of our included population. This limitation is notified in the Discussion section:

“However, our study population consisted mainly of patients who underwent minor elective surgery. This observation should be related to a significant decrease in acute surgical emergencies as observed in previous studies found in the literature [26]. »

5. Please revise well and do specify/define/limit your "surgery" which is different from the perception of "surgery" of many readers.

As suggested by the reviewer, we have specified the type of surgery included throughout the manuscript: mainly minor and elective. In addition, we specified in the Methods part and in the Limits part that neither cardiac, vascular or neurological surgery was included in our study.

6. After clarification of "surgery", further review can be proceeded.

Dear editor, thank you for your comments which greatly improve the relevance and applicability of this manuscript.
